# Suppression of Adipocyte Differentiation by Foenumoside B from *Lysimachia foenum-graecum* Is Mediated by PPARγ Antagonism

**DOI:** 10.1371/journal.pone.0155432

**Published:** 2016-05-13

**Authors:** Hyun Jeong Kwak, Hye-Eun Choi, Jinsun Jang, Soo Kyoung Park, Byoung Heon Cho, Seul Ki Kim, Sunyi Lee, Nam Sook Kang, Hyae Gyeong Cheon

**Affiliations:** 1 Department of Pharmacology, Gachon University School of Medicine, Incheon, Republic of Korea; 2 Natural Substance Research Team, Pharmaceutical R&D center, Kolmar Korea Co. Ltd., Sejong, Republic of Korea; 3 Department of Systems Biology, Sookmyung Women’s University, Seoul, Republic of Korea; 4 Graduate School of New Drug Discovery and Development, Chungnam National University, Daejeon, Republic of Korea; 5 Gachon Medical Research Institute, Gil Medical Center, Incheon, Republic of Korea; East Tennessee State University, UNITED STATES

## Abstract

*Lysimachia foenum-graecum* extract (LFE) and its active component foenumoside B (FSB) have been shown to inhibit adipocyte differentiation, but their mechanisms were poorly defined. Here, we investigated the molecular mechanisms responsible for their anti-adipogenic effects. Both LFE and FSB inhibited the differentiation of 3T3-L1 preadipocytes induced by peroxisome proliferator-activated receptor-γ (PPARγ) agonists, accompanied by reductions in the expressions of the lipogenic genes aP2, CD36, and FAS. Moreover, LFE and FSB inhibited PPARγ transactivation activity with IC_50_s of 22.5 μg/ml and 7.63 μg/ml, respectively, and showed selectivity against PPARα and PPARδ. Rosiglitazone-induced interaction between PPARγ ligand binding domain (LBD) and coactivator SRC-1 was blocked by LFE or FSB, whereas reduced NCoR-1 binding to PPARγ by rosiglitazone was reversed in the presence of LFE or FSB. *In vivo* administration of LFE into either ob/ob mice or KKAy mice reduced body weights, and levels of PPARγ and C/EBPα in fat tissues. Furthermore, insulin resistance was ameliorated by LFE treatment, with reduced adipose tissue inflammation and hepatic steatosis. Thus, LFE and FSB were found to act as PPARγ antagonists that improve insulin sensitivity and metabolic profiles. We propose that LFE and its active component FSB offer a new therapeutic strategy for metabolic disorders including obesity and insulin resistance.

## Introduction

*Lysimachia foenum-graecum* extract (LFE) has been used as a traditional oriental medicine to treat various diseases, such as, colds, rheumatism, headaches, toothaches, and digestive dysfunctions [1, 2]. However, the active component responsible for these wide ranging pharmacological properties has not been identified. Nevertheless, anti-oxidant effects have been associated with phenolics and flavonoids in LFE, and one of its triterpene glycosides, foenumoside E has been reported to have anti-inflammatory effects [2]. Recently, LFE was found to have anti-adipogenic effects by high throughput screening of natural product extract library, and FSB was found to be the active component responsible for the inhibitory effects of LFE during adipocyte differentiation [3, 4]. However, how FSB suppresses adipocyte differentiation at the molecular level was not determined.

Adipocyte differentiation is critical for energy and endocrine homeostasis and is a multi-step process that requires the strict control of several transcription factors [5–7]. Peroxisome proliferator-activated receptor-γ (PPARγ) is a member of the nuclear receptor superfamily of ligand-activated transcription factors, and regulates glucose and lipid homeostasis [8, 9]. PPARγ is also a master regulator of adipocyte differentiation, which is achieved by modulating gene transcription resulting from the recruitments of various transcriptional coactivators and corepressors. Moreover, specific interactions between these coactivators and PPARγ cause differential effects in response to a variety of their ligands. Members of the p160 family, such as, steroid receptor coactivator-1 (SRC-1) [10], transcriptional intermediatory factor-2, and TRAP/DRIP [11] are known to interact directly with PPARγ. On the other hand, nuclear receptor corepressors such as NCoR downregulated PPARγ-mediated transcriptional activity [12]. The other master regulator gene that determines adipocyte differentiation is C/EBPα (CCAAT/enhancer-binding protein-α), which acts to maintain PPARγ expression and promote adipogenesis in association with PPARγ [13, 14].

The PPARγ agonists rosiglitazone and pioglitazone were developed as insulin sensitizers to treat type 2 diabetes mellitus. However, when PPARγ agonists were used clinically, unwanted side effects, including weight gain, were reported, possibly because of the hyperactivation of PPARγ [15, 16]. In addition, PPARγ agonists were associated with the development of hepatic steatosis in rodents [17], whereas several PPARγ antagonists were shown to ameliorate insulin resistance and hepatic steatosis, accompanied by reduced body weights [18, 19]. However, the effects of PPARγ activation on insulin resistance produced inconsistent results. Heterozygous PPARγ deficient mice display improved insulin resistance and dyslipidemia induced by a high-fat diet, but body weights similar to mice on a normal diet [15, 16, 20]. On the contrary, gradual reduction of PPARγ as well as PPARγ mutation resulted in insulin resistance, in association with lipodystrophy [21, 22]. Thus, the extent of PPARγ activation may produce differential effects with regard to the treatment of metabolic disorders.

Based on previous findings that LFE and FSB exhibit anti-adipogenic effects, and that PPARγ plays a key role in adipocyte differentiation, we investigated whether PPARγ antagonism is responsible for the anti-adipogenic actions of LFE and FSB. We further extended our research to examine the *in vivo* effects of LFE using ob/ob mice and KKAy mice, both of which are well-known models of metabolic diseases.

## Materials and Methods

### Chemicals

Dulbecco's modified Eagle's medium (DMEM) containing low or high glucose levels, fetal bovine serum (FBS), fetal calf serum (FCS), penicillin, and streptomycin were obtained from GIBCO (Grand Island, NY). Antibodies against C/EBPα, PPARγ, and β-actin were from Santa Cruz Biotechnology (Santa Cruz, CA). The RNA extraction kit was from Intron Biotechnology (Seoul, Korea). PPARγ, aP2, CD36, FAS, LPL, and GAPDH oligonucleotide primers were from Bioneer Co. (Daejeon, Korea). Rosiglitazone, pioglitazone, GW0742, GW7647, protein inhibitor cocktail, phenylmethyl sulfonylfluoride, hematoxylin,eosin and all other chemicals were from Sigma (St. Louis, MO). LFE and FSB were isolated from *Lysimachia foenum-graecum* as previously described [3, 4].

### Animals

Male ob/ob mice (5 weeks old) were purchased from the Korea Research Institute of Bioscience and Biotechnology (Ochang, Korea). Male KKAy mice (5 weeks old) were purchased from CLEA (Tokyo, Japan). Animals were acclimated for one week and maintained under constant conditions (temperature: 20 ± 2°C, humidity: 40–60%, light/dark cycle: 12 h) for 8 weeks or more. For experiments, mice were divided into three groups (n = 10 each group): Group 1 (the control group) animals were treated with vehicle solution containing 0.5% CMC; Group 2 treated with LFE (100 mg/kg); Group 3 treated with LFE (300 mg/kg) by oral gavage once daily for 8 weeks. Body weights, food intake and blood glucose were measured weekly at the same time every week (between 10:00 and 11:00 AM) for 8 weeks. Food intake was determined by measuring the difference between the preweighed original amount of food and the weight of the food left in each cage (n = 5 in each cage). Blood glucose concentrations were determined in tail vein using Allmedicus Gluco Dr. Plus (Seoul, Korea) in fed state. All animal procedures were performed in accordance with the Guide for the Care and Use of Laboratory Animals published by the US National Institute of Health (NIH Publication No. 8523, revised 2011) and approved by the Animal Care and Use Committee of Gachon University.

### Cell culture and adipocyte differentiation

The 3T3-L1 preadipocytes and HEK293T cells were obtained from the Korean Cell Line Bank (Seoul, Korea). Cells were grown in DMEM-high glucose (3T3-L1) or DMEM-low glucose (HEK293T) containing 10% heat-inactivated FBS, penicillin (100 units/ml) and streptomycin sulfate (100 μg/ml) in a humidified 5% CO_2_ atmosphere at 37°C. To induce adipocyte differentiation, 3T3-L1 cells were cultured until 100% confluent, and then maintained in culture medium for 2 days. Differentiation was induced by exposing cells to either rosiglitazone (50 μM) or pioglitazone (10 μM) in DMEM containing 10% FBS and 1 μg/ml insulin for 2 days in the presence or absence of the indicated concentrations of LFE or FSB. Medium were then replaced with DMEM-high glucose without rosiglitazone or pioglitazone but in the presence or absence of LFE or FSB. Media were then changed every two days for 6 days.

### Oil Red O staining

Differentiated 3T3-L1 cells were fixed with 10% formalin for 10 min and then stained with 0.3% Oil Red O/60% isopropanol solution for 20 min, which was then removed by washing with distilled water. Lipid droplets were visualized and images were captured by phase contrast microscopy. To quantify cellular triglyceride (TG) levels, stained cells were eluted with isopropanol and OD values were determined using a Perkin Elmer VictorX4 (Waltham, MA) at 490 nm.

### Western blot analysis

Cells were harvested in PRO-PREP^™^ protein extraction solution (Intron Biotechnology) and incubated for 30 min at 4°C. Cell debris was removed by microcentrifugation, and supernatants containing proteins were collected. Protein concentrations were determined using Bio-Rad protein assay reagent, according to the manufacturer's instructions. Briefly, proteins (30 μg) were separated by 10% SDS-polyacrylamide gel electrophoresis (SDS-PAGE), and protein samples were transferred to PVDF membranes. Blots were incubated overnight with blocking solution (4% BSA) at 4°C, incubated overnight with a primary antibody against PPARγ and C/EBPα, washed four times with Tween 20/Tris-buffered saline (T-TBS), and incubated with horseradish peroxidase-conjugated secondary antibody (1:1000) for 1 h at room temperature. Blots were then washed three times with T-TBS, and developed using an enhanced chemiluminescence kit (Amersham Life Science, Buckinghamshire, UK).

### RNA Preparation and Real-time PCR

Total cellular RNA was isolated using Easy Blue^®^ kits (Intron Biotechnology). One μg of RNA per sample was reverse transcribed using ReverTra Ace qPCR RT master mix (Toyobo, Japan). Quantitative real-time PCR (qPCR) was performed using incorporation of SYBR green (Toyobo, Japan). The mouse primers used were as follows; PPARγ: 5′-CAT CCA AGA CAA CCT GCT GC-3′ (F) and 5′-TGT GACGAT CTG CCT GAG GT-3′ (R), aP2: 5′-ATT TCC TTC AAA CTG GGC GT-3′ (F) and 5′-GGT CGA CTT TCC ATC CCA CT-3′ (R), CD36: 5′-ATG ACG TGG CAA AGA ACA GC T-3′ (F) and 5′-AAG GCT CAA AGA TGG CTC C-3′ (R), fatty acid synthase (FAS): 5′-TAC TGC GAT TTC TCC TGG CTG-3′ (F) and 5′-AAC CAT AGG CGA TTT CTG GG-3′ (R), lipoprotein lipase (LPL): 5′-CGC TCT CAG ATG CCC TAC AA-3′ (F) and 5′-GAG AAA TCT CGA AGG CCT GG-3′ (R), IL-6: 5′-GAG GAT ACC ACT CCC AAC AGA CC (F) and 5′-AAG TGC ATC ATC GTT GTT CAT AC (R), IL-1β: 5′-CAG GAT CAG GAC ATG AGC ACC (F) and 5′-CTC TGC AGA CTC AAA CTC CAC (R), TNF-α: 5′-ATG AGC ACA GAA AGC ATG AT -3′ (F) and 5′-TAC AGG CTT GTC ACT CGA AT -3′ (R), iNOS: 5′-GGT GTT GAA GGC GTA GCT GA -3′ (F) and 5′-ATC ATG GAC CAC CAC ACA AGC -3′ (R),GAPDH: 5′-TTC ACC ACC ATG GAG AAG GC-3′ (F) and 5′-GGC ATG GAC TGT GGT CAT GA-3′ (R). The mRNA levels were determined using a Roche Light cycler 2.0 (Roche Bio Inc., Bazel, Switzerland). Results were expressed as ratios versus GAPDH.

### Cell transactivation and mammalian two-hybrid assay

pFA-Gal4-PPARα-LBD, pFA-Gal4-PPARδ-LBD, pFA-Gal4-PPARγ-LBD and pM-SRC-1 plasmids were kindly donated by Dr. Young Yang (Sookmyung Women’s University, Seoul, Korea), and pBIND-NCoR-1 by Dr. Jerrold M. Olefsky (University of California, San Diego). pVP-PPARγ and pFR-Luc was kindly provided by Dr. Jung Hyeong Lee (Kangwon National University, Chuncheon, Korea). HEK 293T cells (1.5×10^5^) were seeded in 12-well plates, grown for 24 h, and then transiently co-transfected with pFA-Gal4-PPARα, β, or γ LBD expression vectors and pFR-Luc reporter vector using X-tremeGENE9 DNA transfection reagent (Roche Diagnostics, Mannheim, Germany), according to the manufacturer's instructions. After 16 h of incubation, cells were treated with the indicated concentrations of LFE or FSB in the presence of rosiglitazone (1 μM), GW7647 (a PPARα agonist, 1 μM) or GW0742 (a PPARδ agonist, 1 μM) for 18 h. To perform two-hybrid assay, either coactivator pM-SRC-1 vector or corepressor pBind-NCoR-1 (aa 1803–2439), in the presence of pVP-hPPAR and pFR-Luc reporter vectors, was transiently co-transfected into HEK293T cells using X-tremeGENE9 DNA transfection reagent. After 16 h of incubation, cells were treated with the indicated concentrations of LFE or FSB in the presence or absence of rosiglitazone (1 μM) for 18 h, washed with cold-PBS, lysed, and then, luciferase activity was determined using the Dual luciferase assay system kit (Promega, Madison, WI).

### Molecular docking study between FSB and PPARγ LBD

The docking study using the released crystal complex structure (pdb id: 4Y29^2^) of PPARγ from the RCSB protein databank was carried out. The protein was subjected to the “Prepare protein” with CHAMRm forcefield [23] and default condition, after which PPARγ LBD was defined as a receptor for the docking calculation. And then we defined the expanded binding site based on the original ligand of 4Y29, having 16.77Å binding sphere. To prepare the input molecules, each conformation of FSB was confirmed by Cambridge structure data base (CSD; www.ccdc.cam.ac.uk) and RCSB protein databank site, and then energy minimization was conducted under CHARMm forcefield. Then FSB was docked into the binding site of the PPARγ by the CDOCKER protocol of Discovery Studio4.2. For flexible docking, we selected R357, K358, P359, F360, G361, D362, F363, M364 and E365 in Helix 11 and L452, L453, N454, V455, I456, K457, K458 in Helix 7 residues as flexible residues. Each of the docked conformations was evaluated and ranked using the interaction energy.

### Oral glucose tolerance test (OGTT) and insulin tolerance test (ITT)

OGTT and ITT were conducted after 8 weeks of LFE treatment. For OGTT, mice were fasted for 16 h (from 5:00 PM to 9:00 AM of next day, only water was supplied for all mice) prior to testing and then given an oral injection of d-glucose (2 g/kg). Blood glucose was measured by tail bleeds at the indicated time points for up to 120 min after glucose administration. After 3 days, ITT was done similarly, except the mice were fasted for only 4 h (from 9:00 AM to 1:00 PM, only water was supplied for all mice) and 0.75 IU/kg insulin (Sigma) was administered by *i*.*p*. injection. Blood samples were taken from tail veins at the indicated times for up to 120 min after insulin administration, and blood glucose levels were measured using an Allmedicus Gluco Dr. Plus (Seoul). The area under the curve (AUC) for glucose was calculated for the OGTT or ITT using software OriginPro 6.1 (Origin, Northampton, MA).

### Histopathology

Liver tissues were fixed in 4% paraformaldehyde overnight and embedded in OCT solution. To detect fat deposition, 10 μm liver sections were rinsed with distilled water, stained with H&E or 0.3% Oil Red O in 60% isopropanol for 20 min at 37°C, rinsed with distilled water, and examined under an optical microscope.

### Biochemical analysis

Biochemical parameters were measured using tail blood samples. After centrifugation at 8,000 rpm at 4°C for 5 min, serum levels of TG, alanine aminotransferase (ALT), and aspartate transaminase (AST) were analyzed using commercial kits (Cayman Chemical, Ann Arbor, MI). Hepatic TG was extracted from whole liver homogenates using a modified Folch extraction method [23]. Briefly, lipids were extracted from frozen liver tissues by thawing and homogenizing in a mixture of chloroform, isopropanol, and NP40 (7: 11: 0.1). The tissue homogenates were centrifuged (at 15,000 ×g, 10 min, 4°C) and the resulting supernatants (organic phase) were subject to TG analysis using a kit according to the manufacturer’s instruction. Plasma levels of TNF-α, IL-1β and IL-6 were measured using ELISA kits (Becton Dickinson, Franklin Lakes, NJ).

### Statistical analysis

Results are shown as the means ± SD. Groups were compared by one-way analysis of variance (ANOVA) followed by Tukey’s *post hoc* test. P<0.05 was considered statistically significant. Statistical analysis was performed using the SPSS18.0 software (SPSS Inc., Chicago. IL).

## Results

### Effects of LFE and FSB on PPARγ agonist-induced 3T3-L1 preadipocyte differentiation

**Fig 1:**
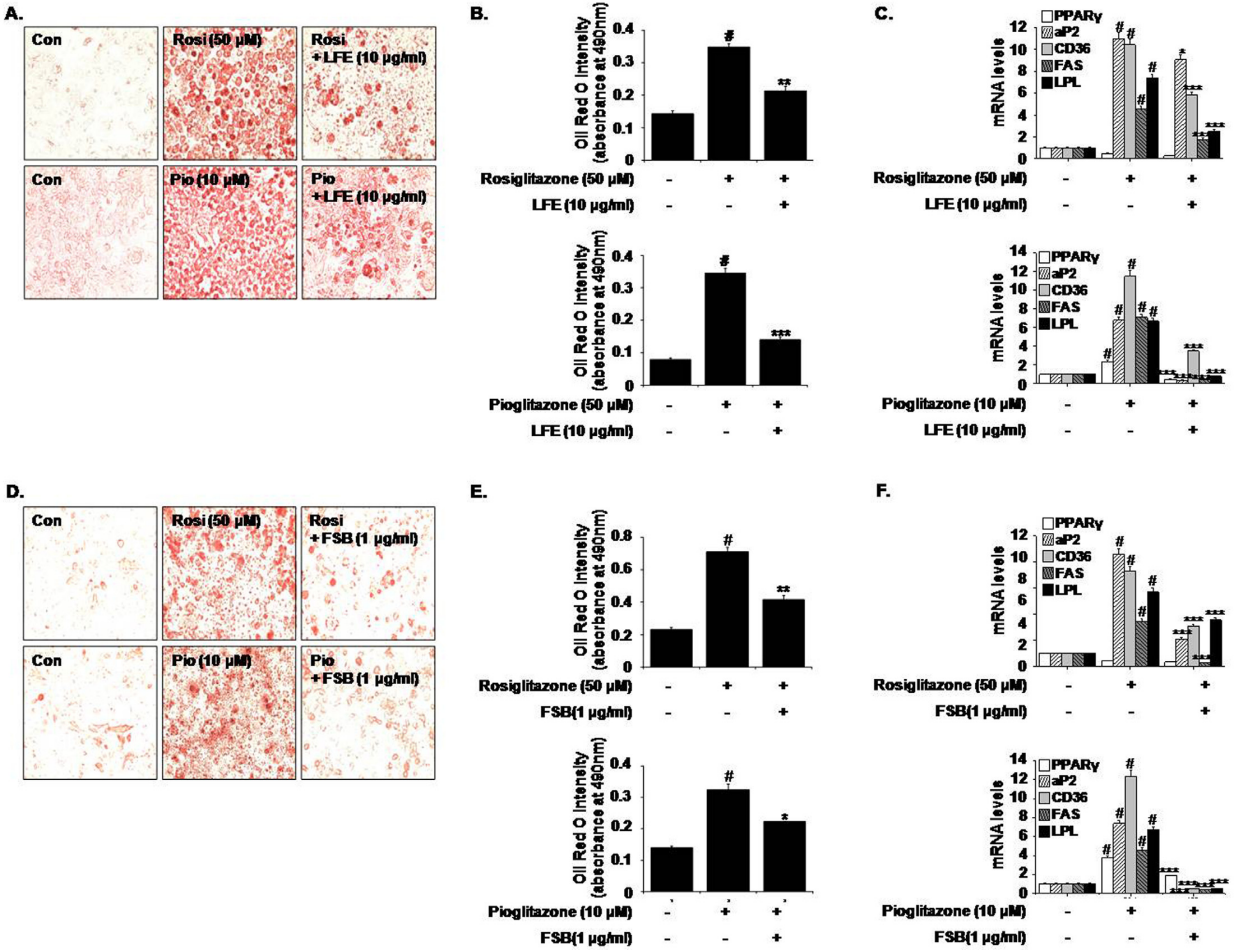
Effects of LFE or FSB on rosiglitazone- and pioglitazone-induced adipocyte differentiation. 3T3-L1 cells were treated with rosiglitazone (50 μM) or pioglitazone (10 μM) in the presence or absence of LFE (10 μg/ml, A-C) or FSB (1 μg/ml, D-F). Six days after the induction of adipocyte differentiation, cells were stained with Oil Red O solution and visualized under an optical microscope (A and D). Absorbance at 490 nm of solutions eluted after Oil Red O staining was used to quantify the extent of adipocyte differentiation (B and E). The mRNA expressions of PPARγ target genes were determined by qPCR (C and F). Experiments were repeated three times in triplicate, and results are presented as means ± SDs. #P<0.05 vs. control; *P<0.05, **P<0.01, ***P<0.001 vs. rosiglitazone or pioglitazone alone.

To determine whether LFE is capable of blocking PPARγ-induced adipocyte differentiation, 3T3-L1 preadipocytes were differentiated in the presence of rosiglitazone (50 μM) or pioglitazone (10 μM), and the effects of LFE were examined. As shown in Fig 1A, LFE (10 μg/ml) reduced accumulations of lipid droplets induced by both PPARγ agonists, and this was confirmed by the reduced absorbance values of eluted Oil Red O solutions (65.3 ± 4.43% reduction in rosiglitazone-induced adipogenesis; 86.6 ± 1.21% reduction in pioglitazone-induced adipogenesis) (Fig 1B). At the same time, the mRNA expressions of PPARγ target genes involved in adiposity and fatty acid metabolism (aP2, CD36, FAS, and LPL), which were markedly increased during PPARγ-induced adipogenesis, were downregulated by LFE as determined by qPCR (Fig 1C), suggesting that LFE antagonizes PPARγ activation. FSB (1 μg/ml) showed similar effects, that is, it inhibited PPARγ-induced adipocyte differentiation (66.3 ± 5.12% reduction in rosiglitazone-induced adipogenesis; 53.3 ± 2.53% reduction in pioglitazone-induced adipogenesis) (Fig 1D and 1E) and target gene expressions (Fig 1F). Neither LFE nor FSB had any cytotoxic effect at the concentrations examined (data not shown).

### Effects of LFE and FSB on PPARγ transactivation

**Fig 2:**
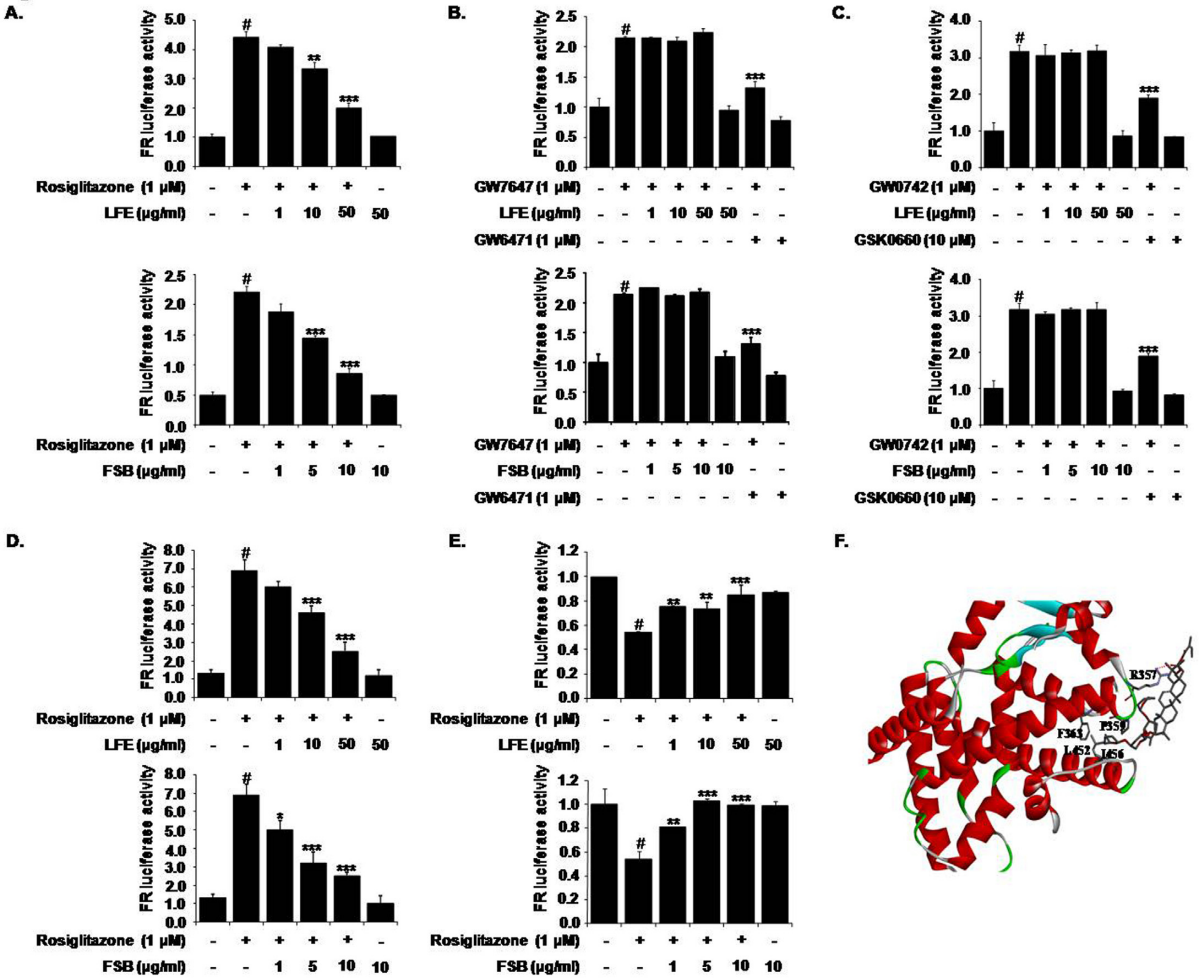
Effects of LFE or FSB on PPARγ transactivation activity and cofactor recruitment. HEK293T cells were transfected with pFA-Gal4-PPARγ-LBD and pFR-Luc reporter vector, and incubated with rosiglitazone (1 μM) with or without LFE or FSB at different concentrations for 18 h. Transactivation activities were assessed by measuring luminescence (A). The effects of LFE or FSB on PPARα and PPARδ were examined after cotransfecting HEK293T cells with pFA-Gal4-PPARα-LBD or pFA-Gal4-PPARδ-LBD. Transactivation was stimulated with the selective PPARα agonist GW7647 or the selective PPARδ agonist GW0742; GW6741 (a selective antagonist of PPARα) and GSK0660 (a selective antagonist of PPARδ) were used as positive controls (B, C). To examine the profiles of cofactor recruitments, HEK293T cells were transiently cotransfected with either pM-SRC-1 (D) or pBind-NCoR-1 (E) together with pVP-PPARγ and pFR-Luc using X-tremeGENE9 DNA transfection reagent. Cells were grown for 24 h in the presence or absence of rosiglitazone with LFE or FSB. The recruitments of the cofactor and corepressor are expressed as fold induction relative to luciferase activity. Experiments were repeated three times in triplicates, and results are presented as means ± SDs. #P<0.05 vs. control; *P<0.05, **P<0.01, ***P<0.001 vs. rosiglitazone alone. The binding mode of FSB on PPARγ was presented (F). FSB was represented gray elementary color. The red line is the hydrogen binding between the side chain of R357 and FSB. The protein molecule is shown as ribbon cartoon except the interacting residues with FSB.

Next, we evaluated the effects of LFE and FSB on PPARγ transactivation activity to explore their action mechanisms with respect to PPARγ antagonism. HEK293T cells were cotransfected with pFA-Gal4-PPARγ-LBD with pFR-Luc, and then incubated with different concentrations of LFE or FSB in the presence or absence of rosiglitazone (1 μM). It was observed rosiglitazone induced PPARγ transactivation and that this activity was inhibited concentration-dependently by LFE or FSB (Fig 2A).

The IC_50_ values of LFE and FSB were estimated to be 22.5 μg/ml and 7.63 μg/ml, respectively, which are somewhat similar to previously reported IC_50_ values (2.5 μg/ml for LFE and 0.2 μg/ml for FSB) as determined using an adipocyte differentiation assay [3, 4]. In contrast to their potent inhibition of PPARγ transactivation, neither LFE nor FSB had any inhibitory effect on PPARα (Fig 2B) or PPARδ transactivation (Fig 2C). The selective PPARα antagonist GW6471 (10 μM) and the PPARδ antagonist GSK0660 (10 μM) were employed as positive controls. These results show that LFE and FSB act as selective PPARγ antagonists.

### Effects of LFE and FSB on coactivator/corepressor recruitments

SRC-1 is a critical transcription coactivator of PPARγ, and it has been previously suggested its interaction with PPARγ could be enhanced by the binding of rosiglitazone to PPARγ LBD [10, 24]. To confirm the PPARγ antagonistic activities of LFE and FSB, we used a mammalian two-hybrid assay. HEK293T cells were co-transfected with pM-SRC-1, pVP-PPARγ and pFR-Luc as a reporter plasmid. As was expected, rosiglitazone enhanced the interaction between PPARγ and SRC-1, and LFE or FSB efficiently antagonized rosiglitazone-stimulated recruitment of SRC-1 to PPARγ in a concentration-dependent manner (Fig 2D), further demonstrating the antagonistic effects of LFE and FSB on PPARγ. The IC_50_ values of LFE and FSB were estimated to be 16.6 μg/ml and 2.85 μg/ml, respectively.

Previously, it was shown that recruitment of NCoR to PPARγ was reduced by PPARγ agonists, and this reduction was reversed in the presence of PPARγ antagonists, leading to the suppression of PPARγ transcriptional activity [12]. As shown in Fig 2E, addition of either LFE or FSB increased the interaction of PPARγ with NCoR-1 (aa 1803–2439), further suggesting that LFE and FSB act as PPARγ antagonists.

### Molecular docking study between FSB and PPARγ

To further validate the binding between PPARγ and FSB, we carried out docking study by CDOCKER [25] interfaced with DiscoveryStudio 4.2 using default parameter comparing with 4Y29 structure [26]. As shown in Fig 2F, FSB bound to the PPARγ LBD. The ((2S,6R)-6-methyl-6-methyltetrahydro-2H-pyran-2-yl)oxy group of FSB was positioned the hydrophobic pocket surrounded by side chains of F363, R357 and P359 in Helix 11, and L452 and I456 in Helix 7, which indirectly contributes to the dynamics of AF-2 within PPARγ LBD. Also, guanidium group R357 formed hydrogen bonding with enoate group of FSB. These results are in agreement with PPARγ antagonism of FSB by showing that FSB is able to bind to the PPARγ LBD.

### *In vivo* effects of LFE in ob/ob mice

**Fig 3:**
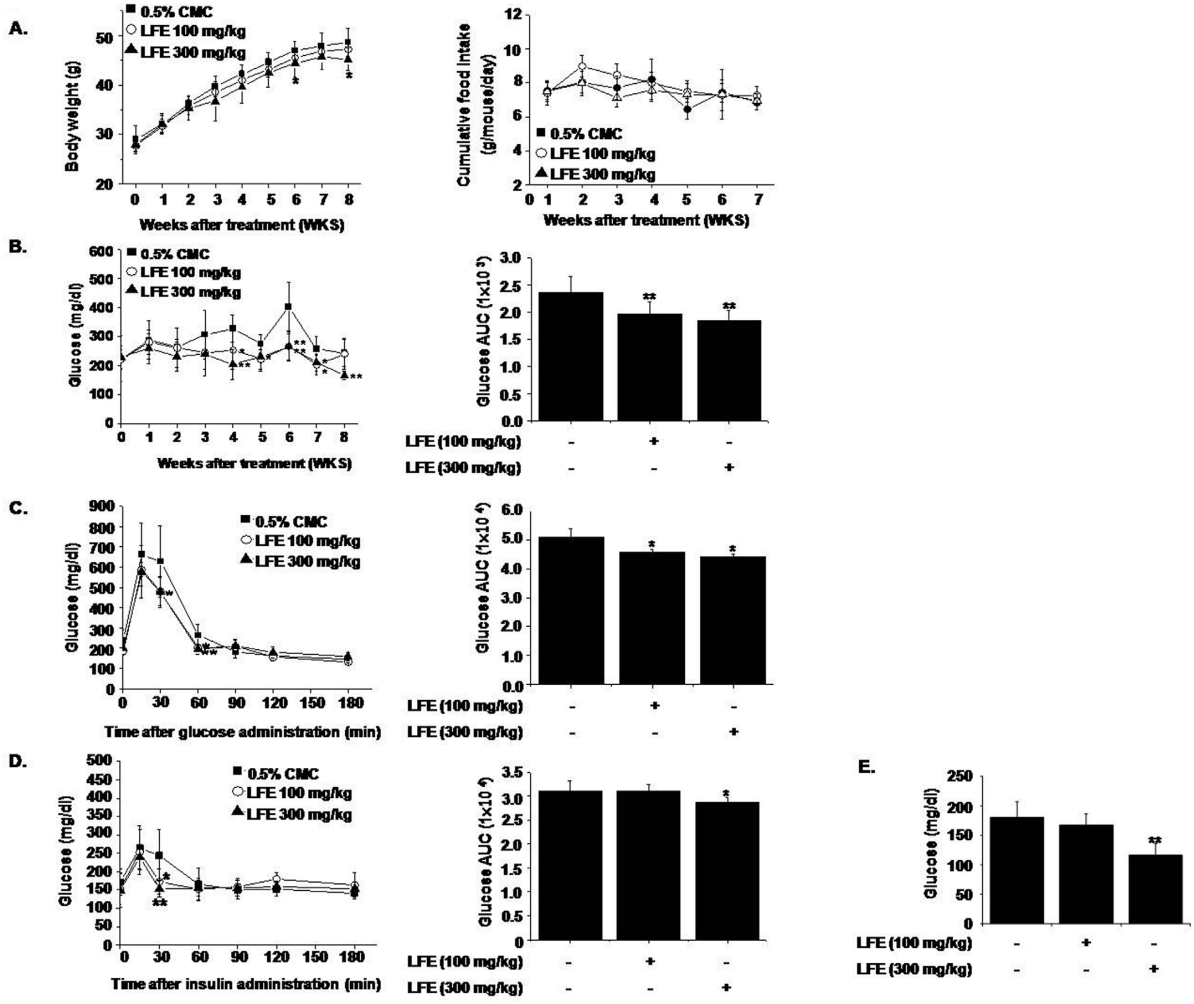
In vivo effects of LFE on ob/ob mice. LFE (100 or 300 mg/kg; n = 10 per group) was orally administered once daily for 8 weeks to 6-week-old ob/ob mice. Body weights (A left), food intakes (A right), and plasma glucose levels (B) were measured weekly. After 8 weeks of treatment, OGTT (C) and ITT (D) were carried out. Fasting plasma glucose levels after 8 weeks treatment were measured (E). Experiments were repeated twice, and results are presented as means ± SDs. *P<0.05, **P<0.01 vs. vehicle (0.05% CMC).

To examine the *in vivo* effects of LFE, we orally administrated LFE (100 or 300 mg/kg) to ob/ob mice once daily for 8 weeks. Starting after 6 weeks of LFE treatment, decreased body weight gains were observed at 300 mg/kg compared with vehicle treatment (7.94 ± 1.54% reduction at 8 weeks), without significant changes in food intake (Fig 3A). As shown in Fig 3B, LFE also lowered blood glucose levels as compared with vehicle controls (AUC analysis showed 21.9 ± 5.54% inhibition at 300 mg/kg LFE), in parallel with improved glucose intolerance as determined by OGTT and ITT (Fig 3C and 3D). Based on AUC analysis of OGTT and ITT curves, inhibition levels at 300 mg/kg LFE were 13.1 ± 1.54% and 7.71 ± 3.03%, respectively. Taken together, these results suggest that LFE improved insulin sensitivity in ob/ob mice and reduced body weight gain.

**Fig 4:**
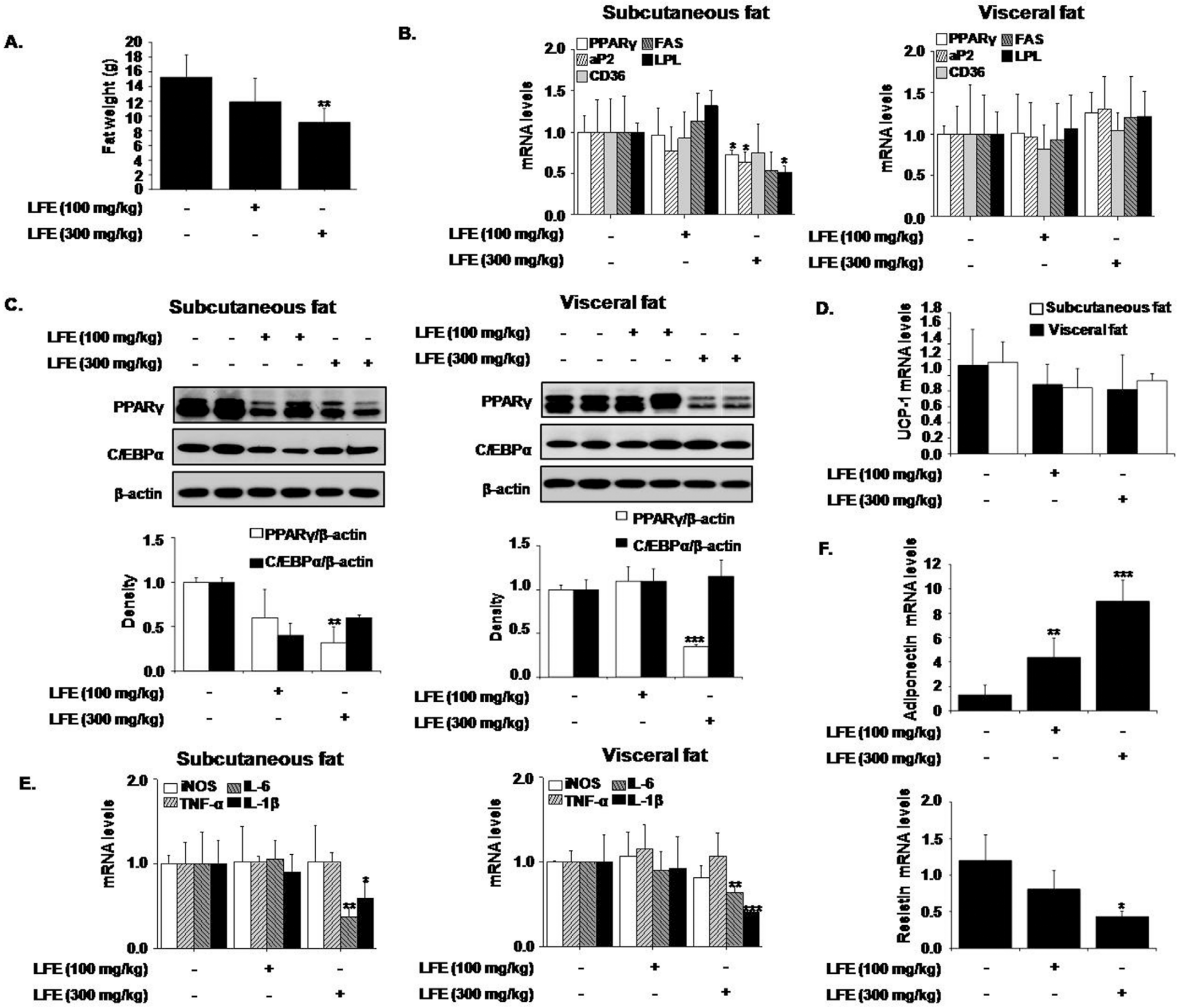
Effects of LFE on fat tissues in ob/ob mice. After 8 weeks of LFE administration, total fat weights were measured (A). The mRNA expressions of PPARγ target genes were determined by qPCR in subcutaneous and visceral fats (B). The protein levels of PPARγ and C/EBPα were detected by western blotting of subcutaneous and visceral fats (C). The mRNA expressions of UCP-1 in subcutaneous and visceral fats were determined by qPCR (D). The mRNA levels of proinflammatory markers, adiponectin and resistin were determined by qPCR (E and F). *P<0.05, **P<0.01, ***P<0.001 vs. vehicle (0.05% CMC).

Next, we examined the effects of LFE on fat tissues isolated from different locations. Weights of subcutaneous and visceral fat tissues were significantly decreased by LFE (59.4 ± 15.4% and 35.9 ± 12.7%, respectively, at 300 mg/kg LFE), and more prominent effects were observed in subcutaneous fat tissues (Fig 4A). However, no weight differences in other tissues, including reproductive tissues, were detected. We also analyzed the mRNA levels of PPARγ target genes in subcutaneous and visceral adipose tissues. In agreement with our *in vitro* results, the mRNA levels of PPARγ, aP2, and LPL in subcutaneous fats were significantly reduced by LFE at 300 mg/kg, and CD36 and FAS mRNA expressions were non-significantly reduced. However, these mRNA expression reductions were not detected in visceral fat (Fig 4B). In addition, LFE treatment reduced the protein levels of PPARγ and C/EBPα, which are both well-known regulators of adipocyte differentiation in subcutaneous fat, but only PPARγ levels decreased in visceral fat (Fig 4C). The expression of UCP-1, a well-known browning marker was unaltered (Fig 4D), suggesting that weight loss by LFE is not likely related with browning effect. Since chronic low-grade inflammation in adipose tissue plays a key role in the development of insulin resistance [27], we examined the effects of LFE and FSB on adipose tissue inflammation. LFE treatment into ob/ob mice reduced the mRNA expression of IL-6 and IL-1β in subcutaneous and visceral fats (Fig 4E). In addition, mRNA expressions of resistin decreased with increased adiponectin mRNA expression by LFE administration, further supports the insulin sensitizing effects of LFE. Together these results indicated LFE reduced body weight gains and improved glucose intolerance, possibly via PPARγ antagonism, and that its effects were more significant in subcutaneous adipose tissues.

**Fig 5:**
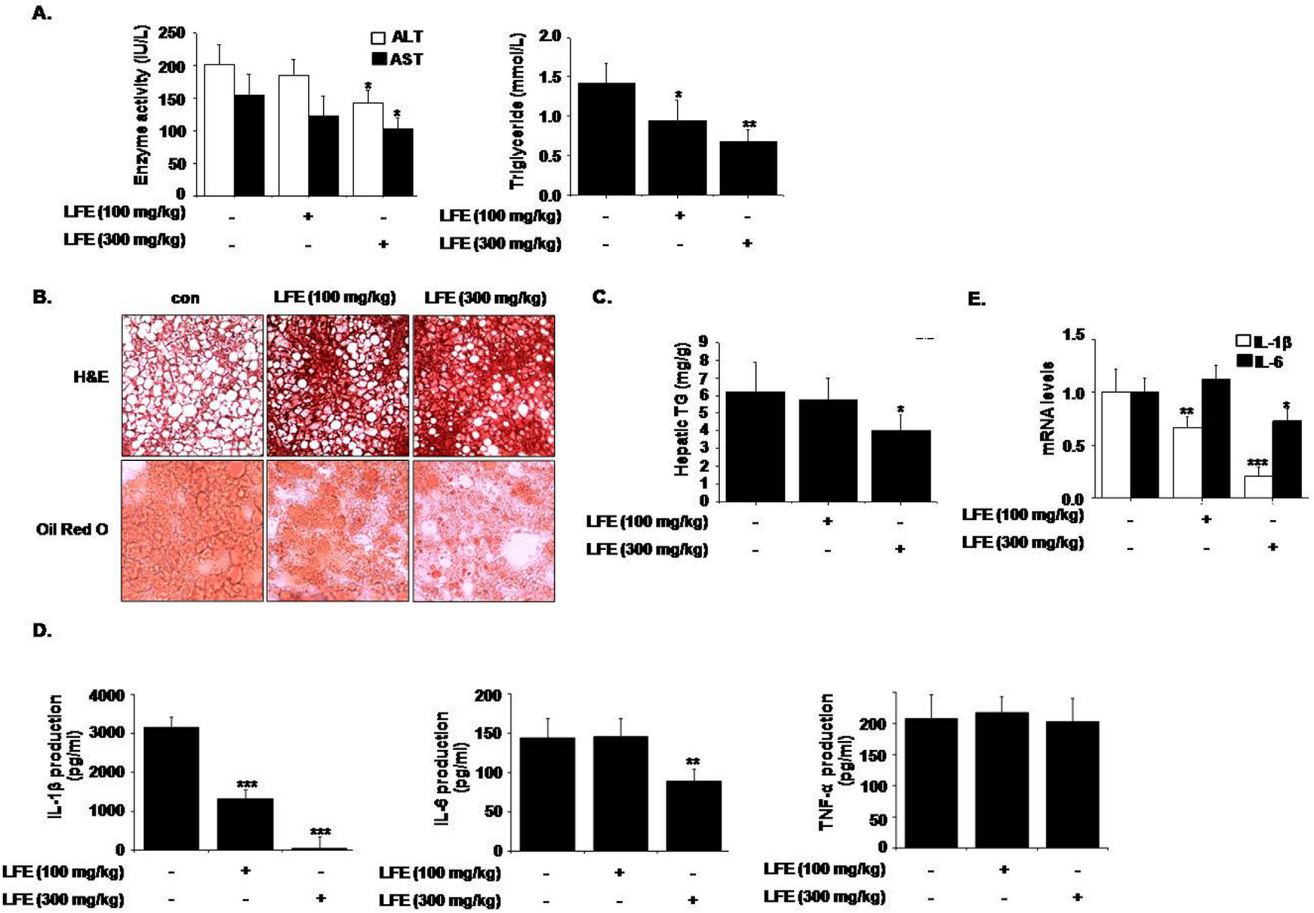
Effects of LFE on hepatic steatosis in ob/ob mice. After 8 weeks of LFE administration, plasma levels of ALT and AST, and TG were determined using commercial kits (A). Liver tissues were frozen and tissue sections were stained with either H&E or Oil Red O, and visualized under an optical microscope (B). Hepatic TG contents were measured using commercial kit (C). Plasma levels of proinflammatory cytokines (IL-1β, IL-6, and TNF-α) were measured using ELISA kits (D). Hepatic mRNA levels of IL-1β and IL-6 were measured (E). *P<0.05, **P<0.01, ***P<0.001 vs. vehicle (0.05% CMC).

In addition, we examined whether LFE had a beneficial effect on hepatic steatosis. LFE (300 mg/kg) lowered serum AST and ALT levels (by 28.9 ± 8.54% and 33.9 ± 9.35%, respectively) and TG levels (by 52.1 ± 12.1%) (Fig 5A). Lipid accumulation in liver was also attenuated by LFE as determined by H&E and Oil Red O staining (Fig 5B). The hepatic TG content was significantly reduced by LFE treatment relative to vehicle control (by 35.4 ± 8.94% reduction at 300 mg/kg LFE) (Fig 5C). Since insulin resistance and metabolic dysfunction are closely related to inflammatory status, we checked proinflammatory cytokine levels in plasma, and found plasma levels of IL-1β and IL-6 were reduced by LFE treatment (300 mg/kg) (by 98.2 ± 12.0% and 35.3 ± 8.96%, respectively) (Fig 5D). Moreover, hepatic mRNA levels of IL-1β and IL-6 were also reduced by LFE (300 mg/kg) (by 79.2 ± 8.18% and 27.1 ± 11.0%, respectively) (Fig 5E). These results are in agreement with previous reports of the anti-inflammatory effects of LFE [2]. However, LFE had no effect on plasma TNF-α. Thus, it appears the anti-inflammatory activities of LFE may also contribute to its beneficial effect on hepatic steatosis.

### *In vivo* effects of LFE in KKAy mice

**Fig 6:**
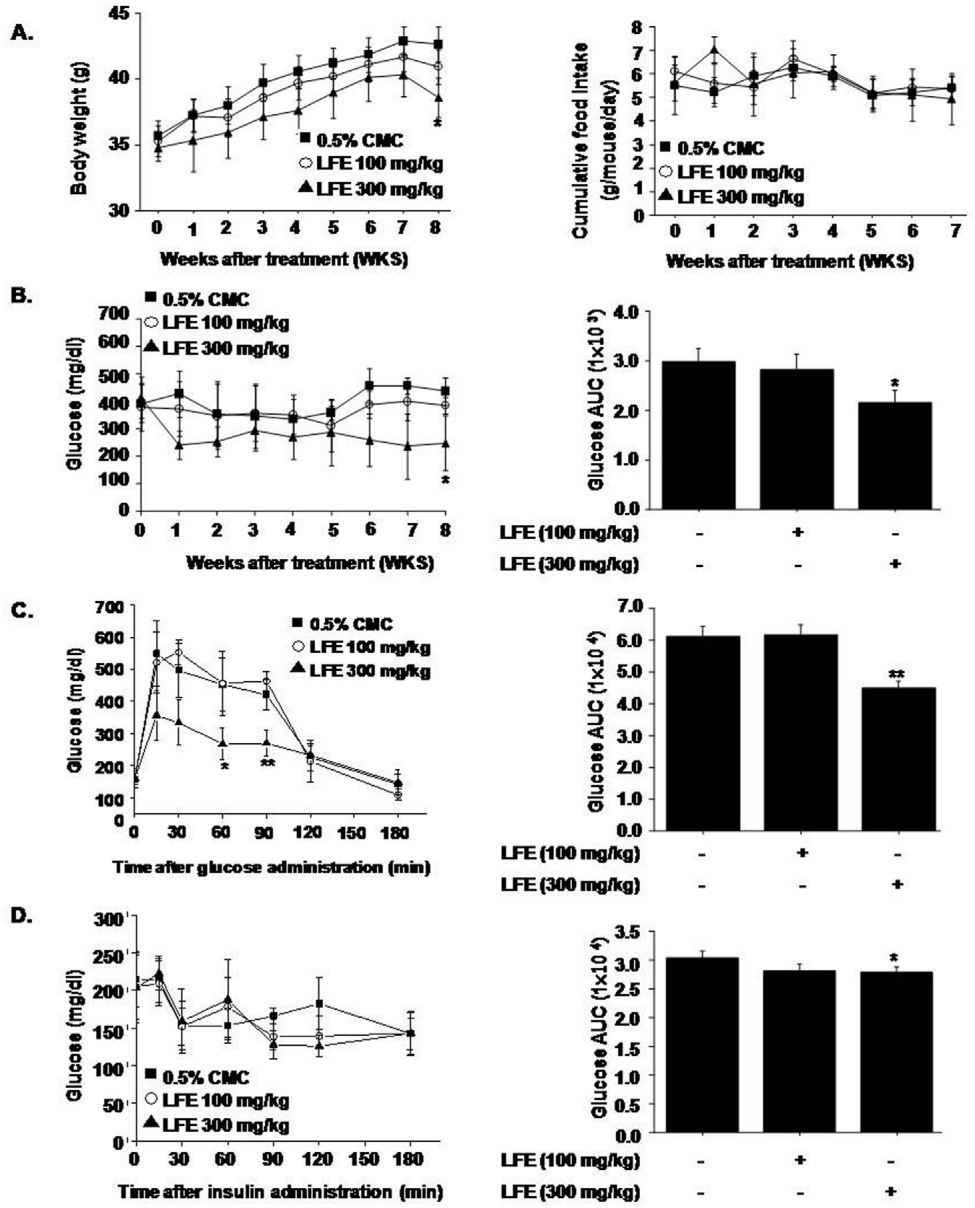
In vivo effects of LFE on KKAy mice. LFE (100 mg/kg, 300 mg/kg; n = 10 per group) was administered once daily for 8 weeks to 6-week-old KKAy mice. Body weights (A left), food intakes (A right), and plasma glucose levels (B) were measured weekly. After 8 weeks of treatment, OGTT (C) and ITT (D) were carried out. Experiments were repeated twice, and results are presented as means ± SDs. *P<0.05, **P<0.01 vs. vehicle (0.05% CMC).

**Fig 7:**
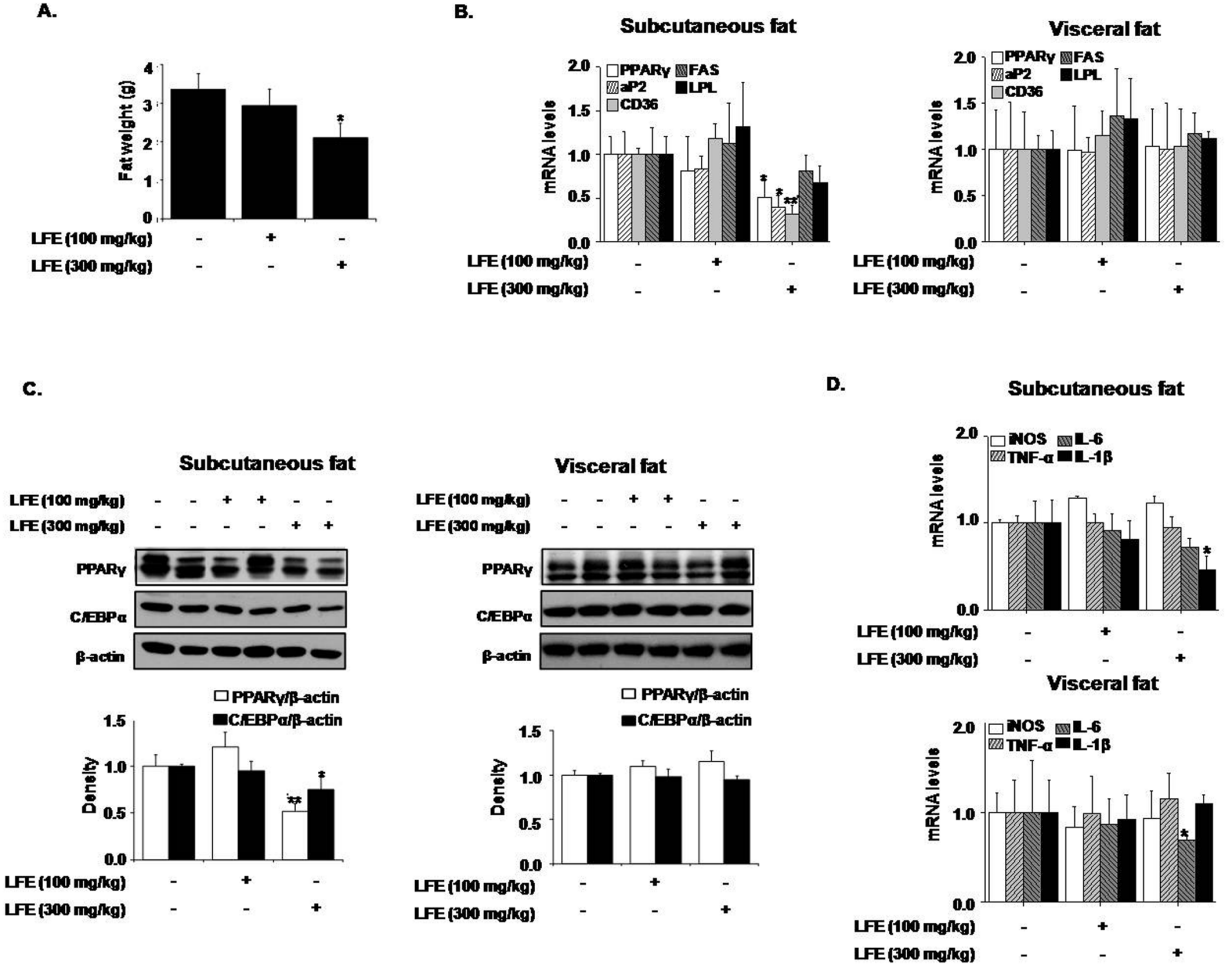
Effects of LFE on fat tissues in KKAy mice. After 8 weeks of LFE administration, total fat weights were measured (A). The mRNA expressions of PPARγ target genes were determined by qPCR in subcutaneous and visceral fats (B). The protein levels of PPARγ and C/EBPα were detected by western blotting of subcutaneous and visceral fats (C). The mRNA levels of proinflammatory markers were determined by qPCR in subcutaneous and visceral fats (D). *P<0.05, **P<0.01 vs. vehicle (0.05% CMC).

**Fig 8:**
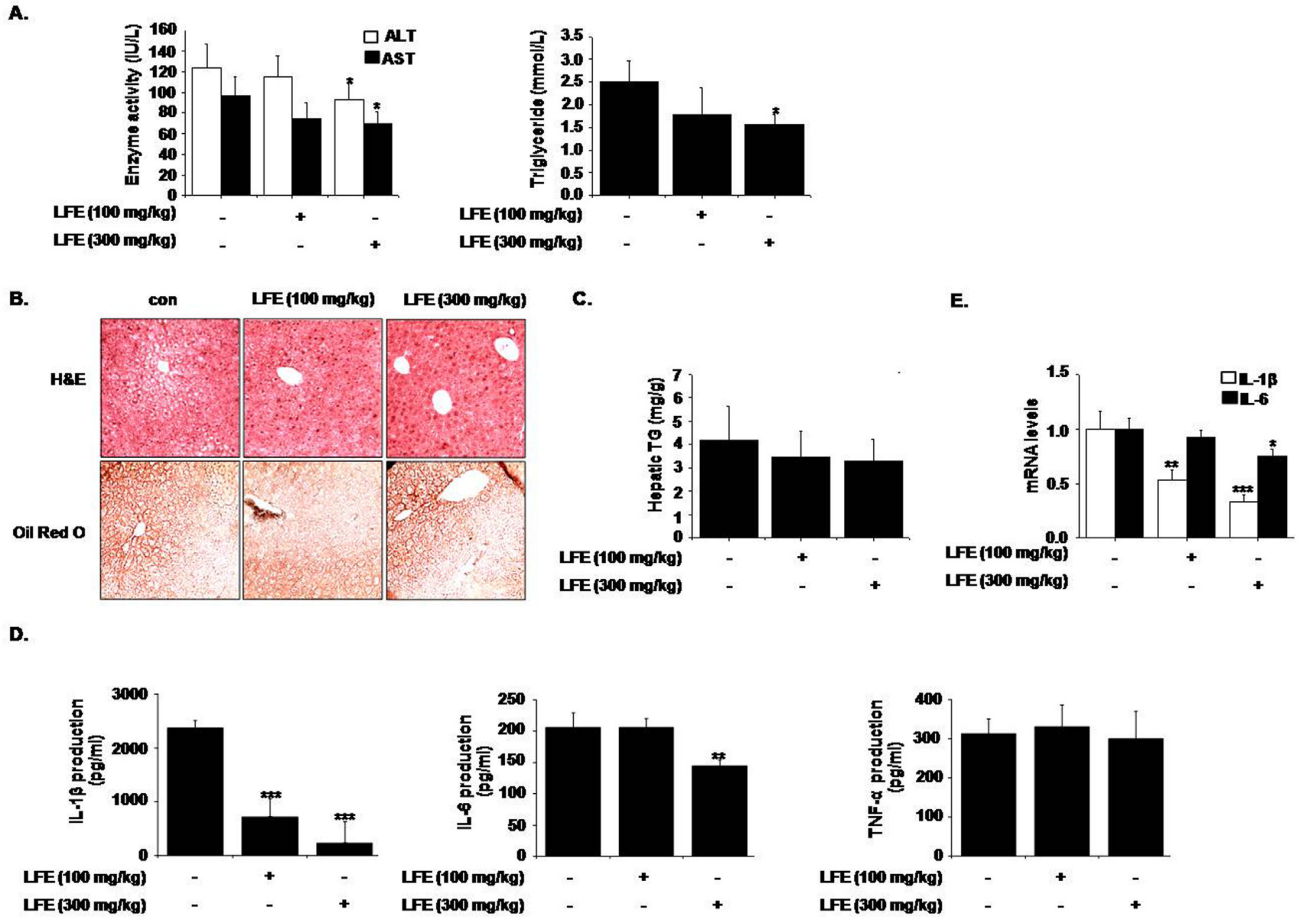
Effects of LFE on hepatic steatosis in KKAy mice. After 8 weeks of LFE administration, plasma levels of ALT and AST, and TG were determined using commercial kits (A). Liver tissues were frozen and tissue sections were stained with H&E or Oil Red O, and examined under an optical microscope (B). Hepatic TG contents were measured using commercial kit (C). Plasma levels of proinflammatory cytokines (IL-1β, IL-6, and TNF-α) were measured using ELISA kits (D). Hepatic mRNA levels of IL-1β and IL-6 were measured (E). *P<0.05, **P<0.01, ***P<0.001 vs. vehicle (0.05% CMC).

To confirm the beneficial effects of LFE on metabolic disorders, we treated KKAy mice, a heterozygote for the yellow spontaneous mutation (Ay) displaying hyperglycemia, hyperinsulinemia, glucose intolerance, and obesity, with LFE for 8 weeks. The results obtained concurred with those observed in ob/ob mice. LFE (at 300 mg/kg daily) reduced body weight gain (9.46 ± 3.26%) and plasma glucose levels (27.5 ± 6.59%), and improved insulin resistance (26.4 ± 3.52% reduction in OGTT AUC; 8.07 ± 2 69% reduction in ITT AUC) (Fig 6A–6D). Consistent with the obtained results from ob/ob mice, mRNA levels of PPARγ, aP2, LPL, IL-6 and IL-1β, and protein levels of PPARγ and C/EBPα were reduced in subcutaneous fat by LFE at this dosage (Fig 7A–7D). In particular, LFE treatment was found to markedly improve liver dysfunction, as assessed by serum analysis (ALT, AST and TG levels) and microscopic observations after H&E and Oil Red O staining (Fig 8A and 8B). No obvious phenotype of fatty liver was observed, in parallel with reduced hepatic TG content compared with vehicle treatment although statistically insignificant (Fig 8C). Additionally, plasma levels of IL-1β and IL-6 (90.0 ± 18.2% and 30.1 ± 4.28% inhibition, respectively) as well as their hepatic mRNA levels (66.5 ± 6.23% and 24.9 ± 6.60% inhibition, respectively) were also reduced by LFE (300 mg/kg) (Fig 8D and 8E).

## Discussion

Present study demonstrates that LFE and its active component FSB act as selective PPARγ antagonists and reduce body weights and hepatic steatosis, and improve insulin resistance in ob/ob mice and KKAy mice. In particular, both suppressed the adipocyte differentiation of 3T3-L1 cells via selective PPARγ antagonism but had little effect on PPARα or PPARδ. Evidence that LFE and FSB are PPARγ antagonists is based on the results of the PPARγ-driven adipocyte differentiation and luciferase assay, and the mammalian two-hybrid assay, all support their key action mechanism involves selective PPARγ antagonism.

PPARγ is a master regulator of adipocyte differentiation, and its activation is necessary and sufficient for adipogenesis. It has been shown the ectopic expression and activation of PPARγ in fibroblasts are sufficient to induce an adipogenic response [28], and that PPARγ is involved in adipogenesis *in vivo* in genetically manipulated chimeric mice [29]. Furthermore, PPARγ plays important roles in glucose and lipid homeostasis, and several PPARγ agonists have been developed to ameliorate imbalances in glucose and lipid metabolism [8, 9]. However, increased adipocyte differentiation leads to excessive TG accumulation, and chronic hyperactivation of PPARγ appears to be associated with increased body weight as has been reported for rosiglitazone [15, 16]. Therefore, moderate degrees of PPARγ blockade may provide better metabolic profiles by reducing obesity. In support of this, partial inactivation of PPARγ in PPARγ heterozygotes was found to increase insulin sensitivity, and several pharmacological agents characterized as PPARγ antagonists were shown to ameliorate metabolic disorders [18, 19].

In the present study, LFE and FSB suppressed the adipocyte differentiation of 3T3-L1 cells, and their suppressive effects were associated with marked reductions in PPARγ target genes, such as, aP2, CD36, FAS, and LPL, which are all positively associated with adipogenesis. To confirm the antagonistic effects of LFE and FSB on PPARγ, transactivation assays were conducted in cells cotransfected with PPARγ-LBD plasmid and pFR-Luc reporter vector, and LFE and FSB were observed to concentration dependently inhibit the rosiglitazone-induced transactivation of PPARγ. These results strongly indicate that the LFE- and FSB-induced suppressions of adipocyte differentiation are mediated by PPARγ antagonism. Similar inhibitory effects on adipocyte differentiation have been reported for PPARγ antagonists, such as, BADGE and SR202 [19, 30].

PPARγ ligand binding increases its affinity for a number of coactivators and facilitates chromatin remodeling and target gene transcription [31–33]. The cofactors that interact with PPARγ include SRC-1/NCoA1, TIF2/GRIP1/NCoA2/SRC-2, and pCIP/ACTR/AiB1/SRC-3, and rosiglitazone and pioglitazone induce the recruitments of SRC-1, CBP or PGC-1α when bound to PPARγ [34]. Hence, we further evaluated the recruitment of the representative coactivator SRC-1 to PPARγ LBD after rosiglitazone treatment. Results indicated that both LFE and FSB antagonized the rosiglitazone-induced recruitment of SRC-1 to PPARγ. Further studies to define the profiles of diverse coactivator recruitments would provide better understanding of the action mechanisms of LFE and FSB. In addition, LFE and FSB antagonized the suppressive effects of rosiglitazone on the recruitment of corepressor NCoR-1 to PPARγ, further supporting that both act as PPARγ antagonists [12].

Rosiglitazone, a PPARγ agonist, produced potent insulin sensitizing effects, but at the expense of increased body weight, whereas PPARγ antagonists improve insulin sensitivity without increasing body weight. In the present study, in ob/ob mice, LFE (at 300 mg/kg) not only reduced body weights but also improved glucose tolerance and insulin sensitivity without affecting food intake. Although total weight loss by LFE appears to be mild (~8% reduction), weight reduction is primarily attributed to fat reduction (~37% reduction of fat weights), with especially remarkable reductions in subcutaneous adipose tissue weights. These results suggest LFE attenuates the expressions of PPARγ and its downstream genes more specifically in subcutaneous adipose tissues. The explanation for the relatively selective action of LFE is unclear, but it may be related with the previous findings that PPARγ activation induces increase in subcutaneous fat in human and rodent diabetic models [35, 36]. Previous study on the quantitative chemical analysis showed that the content of FSB, an active component of LFE was about 2.3% of LFE extract [37], and the effective *in vivo* dose in the present study (300 mg/kg) approximated to be 10 mg/kg of FSB, consistent with previous reports [3]. To delineate the mechanism responsible for the glucose lowering effects of LFE *in vivo*, we examined the effects of LFE and FSB on insulin-stimulated glucose uptake in fully differentiated 3T3-L1 adipocytes and in C2C12 differentiated myotubes, and LFE was found to increase insulin-stimulated glucose uptake in C2C12 myotubes, but not in 3T3-L1 adipocytes (results not shown). Further studies are needed to elucidate the reasons for these differential effects and to determine whether PPARγ antagonism by LFE is involved in increased glucose uptake.

Recent studies indicate that elevated adiposity is associated with increased plasma pro-inflammatory cytokine levels [38–40], and insulin resistance is induced by chronic low grade inflammation of adipose tissue, accompanied by infiltration of inflammatory macrophages into adipose tissue [26]. On the other hand, weight loss is known to improve obesity-associated metabolic disorders, particularly chronic low grade inflammation [41]. In the present study, we found that LFE reduced proinflammatory cytokine levels, such as, IL-1β and IL-6, in plasma, subcutaneous and visceral fats and liver tissues in ob/ob mice. Furthermore, oral LFE administration for 8 weeks had beneficial effects on hepatic steatosis in ob/ob mice as evidenced by reduced TG accumulation in livers and improved hepatic morphology, which suggests the anti-inflammatory properties of LFE improve metabolic profiles. However, whether this anti-inflammatory action is linked to PPARγ antagonism needs to be determined. Interestingly, the novel PPARγ antagonist protopanaxatriol isolated from Panax ginseng improved liver steatosis in ob/ob mice [18].

KKAy mice offer another animal model featuring hyperphagia with increased fat mass and insulin resistance due to the presence of the yellow spontaneous mutation (Ay) [42]. LFE (at 300 mg/kg daily) suppressed body weight increases after 8 weeks of treatment, and this was paralleled by reductions in subcutaneous fat weights. Furthermore, improvement on glucose intolerance and insulin sensitivity as well as amelioration of hepatic steatosis was also observed. These results are comparable to those reported previously using high fat diet-induced obese mice [4].

In conclusion, our results demonstrate that LFE and FSB both inhibit PPARγ-stimulated adipocyte differentiation via PPARγ antagonism, and have beneficial effects on body weight, insulin resistance, and hepatic steatosis in ob/ob and KKAy mice. Although we identified PPARγ antagonism as being critical for the mode of action of FSB, additional mechanisms might also be implicated in the anti-adipogenic effects of FSB. Collectively, our results show LFE and FSB are potential therapeutic agents for the treatment of obesity and metabolic diseases.
